# Fibroblast growth factor-2 (FGF2) and syndecan-1 (SDC1) are potential biomarkers for putative circulating CD15+/CD30+ cells in poor outcome Hodgkin lymphoma patients

**DOI:** 10.1186/1756-8722-6-62

**Published:** 2013-08-29

**Authors:** Rajendra Gharbaran, Andre Goy, Takemi Tanaka, Jongwhan Park, Chris Kim, Nafis Hasan, Swathi Vemulapalli, Sreeja Sarojini, Madalina Tuluc, Kip Nalley, Pritish Bhattacharyya, Andrew Pecora, K Stephen Suh

**Affiliations:** 1 John Theurer Cancer Center, Hackensack University Medical Center, D. Jurist Research Building, 40 Prospect Avenue, Hackensack, NJ 07601, USA; 2 Thomas Jefferson University, Philadelphia, PA 19107, USA; 3 Sophic Systems Alliance, Falmouth, MA 02540, USA

**Keywords:** Hodgkin lymphoma, Predictive biomarkers, Relapse, Refractory, Circulating tumor cells, Clinical outcome

## Abstract

**Background:**

High risk, unfavorable classical Hodgkin lymphoma (cHL) includes those patients with primary refractory or early relapse, and progressive disease. To improve the availability of biomarkers for this group of patients, we investigated both tumor biopsies and peripheral blood leukocytes (PBL) of untreated (chemo-naïve, CN) Nodular Sclerosis Classic Hodgkin Lymphoma (NS-cHL) patients for consistent biomarkers that can predict the outcome prior to frontline treatment.

**Methods and materials:**

Bioinformatics data mining was used to generate 151 candidate biomarkers, which were screened against a library of 10 HL cell lines. Expression of FGF2 and SDC1 by CD30+ cells from HL patient samples representing good and poor outcomes were analyzed by qRT-PCR, immunohistochemical (IHC), and immunofluorescence analyses.

**Results:**

To identify predictive HL-specific biomarkers, potential marker genes selected using bioinformatics approaches were screened against HL cell lines and HL patient samples. Fibroblast Growth Factor-2 (FGF2) and Syndecan-1 (SDC1) were overexpressed in all HL cell lines, and the overexpression was HL-specific when compared to 116 non-Hodgkin lymphoma tissues. In the analysis of stratified NS-cHL patient samples, expression of FGF2 and SDC1 were 245 fold and 91 fold higher, respectively, in the poor outcome (PO) group than in the good outcome (GO) group. The PO group exhibited higher expression of the HL marker CD30, the macrophage marker CD68, and metastatic markers TGFβ1 and MMP9 compared to the GO group. This expression signature was confirmed by qualitative immunohistochemical and immunofluorescent data. A Kaplan-Meier analysis indicated that samples in which the CD30+ cells carried an FGF2+/SDC1+ immunophenotype showed shortened survival. Analysis of chemo-naive HL blood samples suggested that in the PO group a subset of CD30+ HL cells had entered the circulation. These cells significantly overexpressed FGF2 and SDC1 compared to the GO group. The PO group showed significant down-regulation of markers for monocytes, T-cells, and B-cells. These expression signatures were eliminated in heavily pretreated patients.

**Conclusion:**

The results suggest that small subsets of circulating CD30+/CD15+ cells expressing FGF2 and SDC1 represent biomarkers that identify NS-cHL patients who will experience a poor outcome (primary refractory and early relapsing).

## Background

Up to 20% of Hodgkin lymphoma (HL) patients are either refractory to treatment (primary refractory) or experience relapse within four years (early relapse) of achieving complete remission (CR), and includes patients who experience progressive disease and patients with a particularly poor prognosis for other reasons [1]. Only half of HL patients survive for two years if front line therapy fails, and autologous hematopoietic stem-cell transplant (ASCT) is only 50% curative [2]. Although the International Prognostic Score was introduced to improve the risk stratification of patients [3], its applicability is limited for predicting high risk cHL patients, regardless of clinical stage. While patients in this group may benefit from analysis of the tumor-associated macrophage marker CD68, which can be used to predict adverse outcomes of cHL [4], the prediction is controversial [5]. The antibody conjugate drug brentuximab vedotin targets CD30. In clinical trials, brentuximab vedotin therapy improved clinical outcomes for relapsing and refractory classical HL (RR-cHL) patients by producing survival times that were 6 months longer than for patients on the conventional treatment arm [6]. This increased survival could perhaps be due to increased chemoresistance that can result from heavy pre-treatment. Therefore, the availability of biomarkers that identify patients who will have a poor outcome to conventional frontline therapy will permit more aggressive treatment of these patients, improving their prognosis.

Classical HL is a monoclonal lymphoid neoplasm that in almost all instances appears to be derived from (post-) germinal center B cells [7-9]. The immunohistochemical (IHC) hallmark of HL tumor cells is CD30 antigen expression [10]. The morphological phenotype of cHL comprises an unusually small number (<2%) of mononuclear Hodgkin (H) cells and multinucleated Reed-Sternberg (RS) cells residing in an extensive inflammatory background, which is mostly composed of T cells, histocytes, eosinophils, plasma cells, and macrophages [10]. This inflammatory background in the tumor microenvironment is maintained by Hodgkin’s and Reed-Sternberg cell (HRS)-derived chemokines and cytokines that recruit the tumor microenvironment cellular components [11-14]. The composition of the tumor microenvironment or the molecular phenotype of the HRS cells, or both, is thought to determine the relative aggressiveness of cHL at an individual level [10].

At presentation, about 10–15% of cHL cases have extranodal involvement [15], which is a negative prognostic factor even for patients with limited stage disease [16]. Extranodal involvement, whether primary or secondary, indicates lymphatic and hematogenous spread of the disease [15]. Therefore, neoplastic HRS cells could reasonably be assumed to occur in peripheral blood, albeit at levels not detectable by present diagnostic techniques, thus resulting in circulating tumor cell (CTC) involvement in HL. CTCs are frequently associated with poor clinical outcomes for solid [17] and liquid tumors [18]. Despite the limited number of cases (about a dozen over the past 100 years) of CTCs in the peripheral blood of HL patients, most were associated with either primary refractory or relapsing disease. In addition, well-established cell lines that have contributed tremendously to the understanding of HL were derived from primary HL tumor cells isolated from extranodal sites: peripheral blood [19], bone marrow [20], or pleural fluid [21] of refractory or relapsing patients. These findings suggest that primary HL tumor cells can escape the physical barrier of the tumor microenvironment into the circulation to access extra-nodal destinations. The limited evidence indicating the presence of HRS cells among peripheral blood leukocytes (PBL) may be a consequence of their low proliferative index, the terminally differentiated status of the RS cells and their lack of mobility, or the propensity of these malignant cells to form a solid tumor mass [22]. These characteristics have hampered investigations aimed at identifying HRS-derived biomarkers in peripheral blood for high risk, poor outcome, primary refractory, and early relapsing cHL patients.

## Results

### Characteristics of clinical samples

The characteristics for clinical samples for PBL are listed in Table 1. Retrospective clinical samples of PBL collected from 25 NS-cHL patients (average age: 34.48 years, range: 20–79, 13 females and 12 males) were categorized into three groups mainly on the basis of their response to frontline therapy: 1) good outcome pre-therapy: chemo-naïve relapse free/progression-free survival > 4 years (GO, n=12); 2) poor outcome pre-therapy: chemo-naïve primary refractory or early relapsing (PO(CN), n=6; 3) poor outcome post-therapy: chemo-exposed, multiple relapse within 4 years (PO(CE), n=7). Among the pre-therapy, chemo-naïve patients (n=18), 68% were diagnosed during early disease stages (I and II), 10% (n=2) at stage III, and 15% (n=3) at stage IV. Of the early stage diagnoses (I and II, n=13), more than 30% (n=4) were either primary refractory or developed early relapses shortly after frontline therapy. The remaining PO(CN) samples were from advanced stages (III & IV). Also, 56% (n=14) of the patients were younger than the average age (34.48 years) at diagnosis.

**Table 1:** Patient characteristics for each clinical outcome group

	**Donors**	**Sex**	**Clinical diagnosis**		**Age**	**Stage at diagnosis**		**Outcome**
			**Subtype**	**Bulky/non-bulky**			**Treatment**	
**Good outcome**	GO1	F	NS	UNSP	29	IV	ABVD	PFS
	GO2	F	NS	B	41	IIA	Stanford V + Rad	PFS
	GO3	F	NS	NB	79	IA	ABVD + Rad	PFS
	GO4	F	NS	B	22	IIA	Stanford V + Rad	PFS
	GO5	M	NS	NB	43	IIA	ABVD	PFS
	GO6	M	NS	B	20	IIA	ABVD+R	PFS
	GO7	F	NS	NB	64	IIA	ABVD	PFS
	GO8	F	NS	B	51	IIIB	ABVD + Rad	PFS
	GO9	F	NS	UNSP	54	IV	ABVD	PFS
	GO10	F	NS	UNSP	22	IIA	ABVD	PFS
	GO11	F	NS	NB	25	IIA	ABVD	PFS
	GO12	F	NS	NB	26	IIB	ABVD	PFS
**Poor outcome (CN)**	PO1	M	NS	UNSP	48	IIA	ABVD+R (1); R+Bendamustine (2) Zevalin(3)	Rel.
	PO2	M	NS	B	24	II	ABVD (1); acc. BEACOPP (4X) std BEACOPP (2X)(2); Bendamustine + R (3); IGEV + Rad (4); BCPAT (5); CR PT	Ref.
	PO3	M	NS	UNSP	25	IIB	ABVD (1); ICE X 3 followed by BPCAT +Local Rad (2); CR PT	Rel.
	PO4	F	NS	B	25	IIA	ABVD (1); ICE (2X) (2); GVD+R+Rad (3); HCVAD 1A (4); F+ECPOCH TH2 Allogenic (5); CR PT	Ref.
	PO5	M	NS	B	49	IV	ABVD + R (1); ICE + R followed by BCPAT (2); R for EBV reactivation (3); CR PT	Ref.
	PO6	M	NS	NB	20	IIIB	ABVD+R (1); ICE *X*2 (2); IGEV+R × 2 (3); Rad (4); BCPAT (5); CR	Ref.
**Poor outcome (CE)**	PO1	M	NS	UNSP	31	IIIB	ABVD (1); ABVD (2); CPPV (3); DICE followed by BPCAT (4); HCVAD 1A + 1B (5); FMPAL (5)	Rel.
	PO2	F	NS	UNSP	23	II	MOPP+ABVD (1); BEAC conditioning pre auto transplant (2); Rad (3); ICE X 2/ESHAP X 6 (4);	Rel.
	PO3	M	NS	UNSP	21	II	ABVD (1); ESHAP x 1 followed by BCPAT (2); Gemcitabine +Navelbine (3); HCVAD X 3A’S followed by FMPAL (4); DLI infusion (5); Revlamid+DLI infusion (6);	Rel.
	PO4	F	NS	B	20	IIA	ABVD+Rad (1); ICE+ auto transplant (2); bone resection+Rad (3); WU protocol phase II Revlamid (4); TH2 Study (EPOCH+FR) NCI protocol followed by BEACOPP pre transplant (5); (No rel.)	Rel.
	PO5	M	NS	UNSP	30	IIB	ABVD + Rad (1); ICE + Gemzar followed by BPCAT (2); ESHAP X 3 (3); HCVAD X 5 followed by FMPAL (4); Bendamustine (SK Protocol) 08–041 (5)	Rel.
	PO6	M	NS	UNSP	49	IIIB	ABVD (1); ICE followed by BCPAT; GVD + R (3); Revlamid (4) SGN-40 × 2 cycles (5); (PD)	Rel.
	PO7	M	NS	UNSP	21	IIA	ABVD (1); ESAHP (2); IGEV (3); BEAC + Rad (4); GDP; R+MOPP (5); died of PD.	Rel.

### Bioinformatics and data mining for potential biomarkers

To enhance the specificity of potential poor outcome biomarkers, a bioinformatics based approach was used. Potential biomarkers for HL were selected from the Cancer Gene Index and screened using a library of HL cell lines. Bioinformatics-guided approaches have the unique advantage of avoiding challenges that arise from the cost, time, and labor that are required to identify potential biomarkers for human diseases. The BioXM software platform (Sophic Alliance, Rockville, MD) was used to mine published data for more than 7,000 cancer genes and 2,200 biomarker genes. These genes were annotated and validated from 18 million Medline abstracts and 24,000 HUGO genes using a combination of algorithmic methods (Biomax Informatics, Munich, Germany), including natural language processing (NPL), Biomarker Role Codes, the NCI Cancer Thesaurus, and Karp’s Evidence Codes [23]. Compilation of the outputs resulted in the identification of 151 candidate HL biomarker genes (Table 2).

**Table 2:** HL-relevant genes identified by bioinformatics data mining


ABCC2	CCR4	CHEK2	ESR2	HSPA8	MALT1	OGG1	SPN
ABL1	CCR7	CLU	EZH2	HYAL2	MLL	PAX5	SRC
ADA	CD14	CNR1	FAS	ICAM1	MME	PDCD1LG2	SST
ADIPOQ	CD2	COL18A1	FCER2	ID2	MPO	PIK3CA	STAT6
AR	CD22	CP	FCGR3A	IFNG	MS4A1	PIM1	TBX21
ATF3	CD27	CR2	FGF2	IGHE	MSH6	PLK1	TERF1
B2M	CD28	CSF3	FHIT	IGLα	MUC16	POU2F2	TRFC
B3GAT1	CD34	CTLA4	FLT3	IL2	MYB	PRL	TGFB1
BCL10	CD38	CXCL10	FSCN1	IL2RA	MYC	PTEN	TIA1
BCL3	CD44	CXCR3	GATA3	IL3	MYOD1	PTH	TNFRSF1β
BCL6	CD46	CXCR4	GFAP	IRF4	NAT2	REL	TNFSF13β
BIC	CD5	CYP17A1	GGT1	ITGA4	NBN	S100A6	TP63
BMI1	CD52	CYP3A43	GHRL	ITGAL	NF1	SDC1	TRAF1
BSG	CD55	D13S25	GPX1	ITGB2	NME1	SELL	TRGα
CASP8	CD59	DUT	HLA-A	JUNB	NOS2	SERPINE1	TSHB
CCL17	CD70	E2F1	HMGB1	LDHA	NPM1	SERPING1	VEGFA
CCL5	CD79A	E2F3	HP	LEP	NPY	SMARCB1	WT1
CCND1	CDC25A	EDN1	HSPA1A	LEPR	NRAS	SOCS1	ZBTB16
CCND3	CDK4	ERBB2	HSPA4	MAL	NTRK2	SPI1	

### The clinical outcome of HL patients is not associated with tumor staging, age, bulkiness or frontline therapy

**Figure 1:**
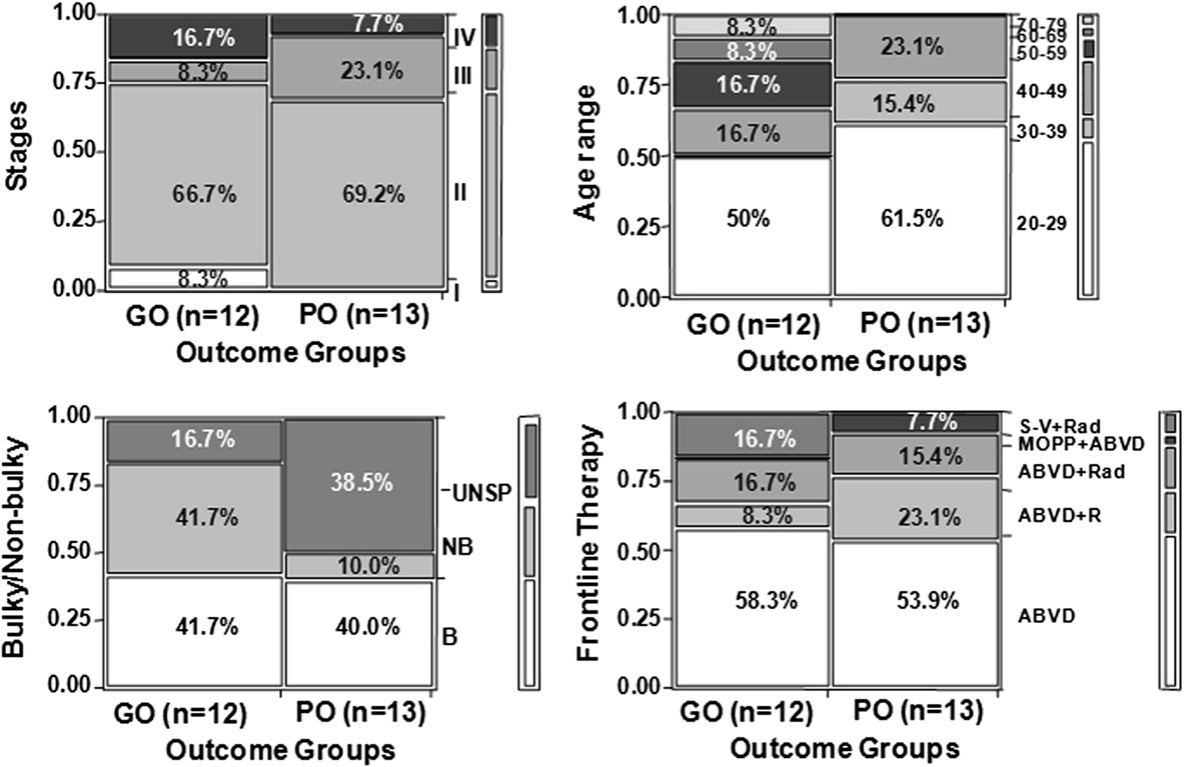
**Lack of association between clinical outcome and tumor staging, age, bulkiness, or frontline therapies and the overexpression of FGF2 and SDC1 by HL cell lines.** Contingency analysis was performed against major clinical characteristics (y-axis, right column) including tumor stage (p > 0.4), age group (p > 0.11), bulkiness of the disease (p > 0.18), and frontline therapies used (p > 0.27) for HL patients with good outcome (GO) vs. poor outcome (PO) (x-axis). The percentage of each clinical characteristic within each group is indicated.

Contingency analyses of 25 NS-cHL patients did not identify associations between clinical outcomes (good outcome (GO), n=12, vs. poor outcome (PO), n=13) and major clinical characteristics such as clinical stage (p > 0.4), age group (p > 0.11), bulky disease (with or without inclusion of unspecified data, p > 0.18), and frontline therapy (p > 0.27) (Figure 1, Table 1). The same analysis of the dataset with the PO(CE) group excluded also failed to identify any relationship between outcome and clinical phenotype. This result differs from established trends used in stratification schemes of current prognostic scoring systems. Our results suggest that alteration of specific molecular signaling may contribute to clinical outcome.

### FGF2 and SDC1 are overexpressed by HL cell lines and by CD30+ cells in the poor outcome group of HL patients

**Figure 2:**
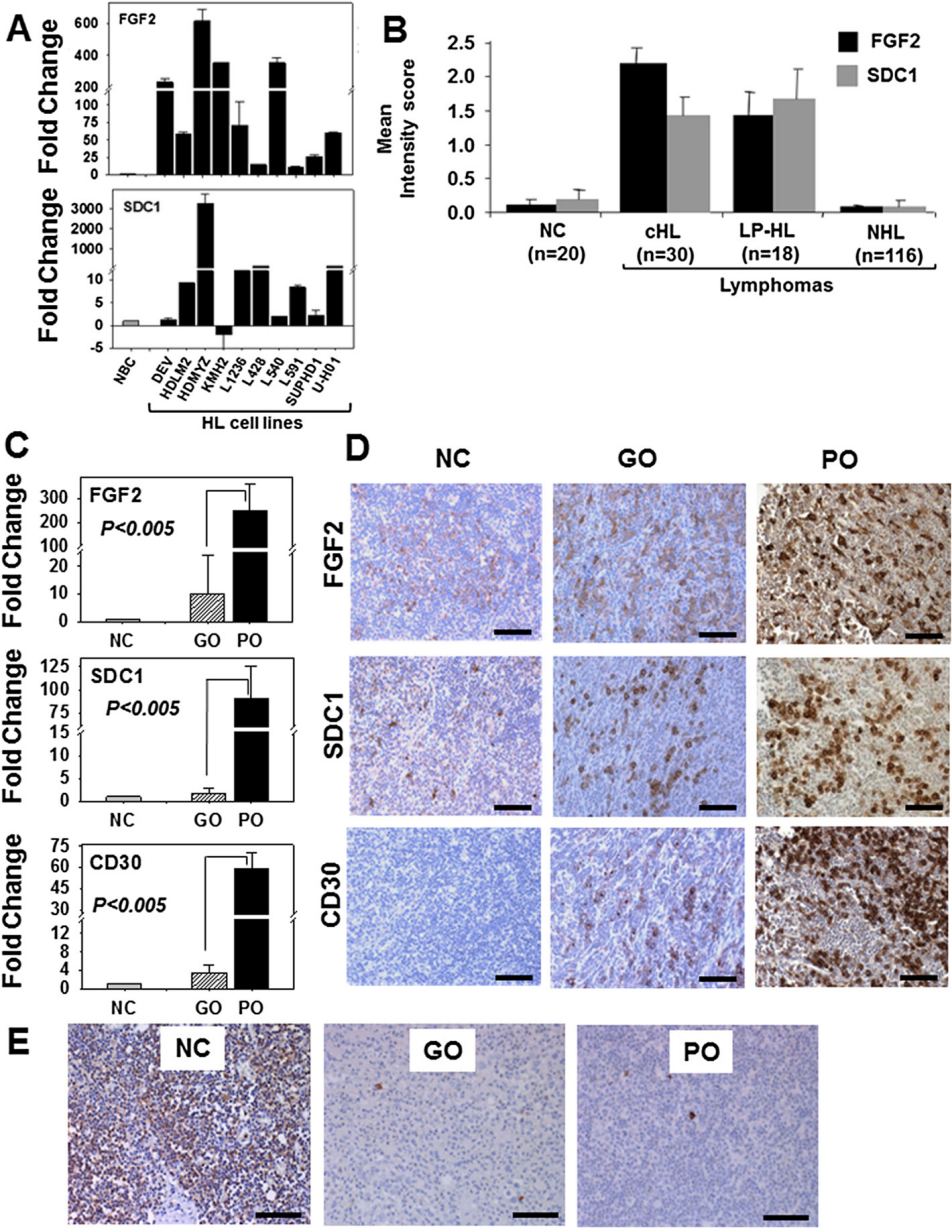
**FGF2 and SDC1 are overexpressed by HL cell lines and by CD30+ cells in the poor outcome HL patient group. (A)** FGF2 and SDC1 expression in 10 different HL cell lines (solid black bar) is represented as the normalized fold change relative to purified normal B-cells (NBC, solid gray bar). The standard error (SE) for each cell line is indicated above each bar. See Table 2 for HL cell line characteristics. **(B)** Qualitative mean intensity scores for FGF2 (solid black bar) and SDC1 (solid gray bar) from immunostained tissues in an array format consisting of 10 normal, 30 classical HL (cHL), and 18 Lymphocyte Predominant-HL (LP-HL) and 116 Non-HL (NHL) samples (y-axis). Immunostaining intensity was scored as 0 (no staining), 1 (weak), 2 (moderate), or 3 (intense). Standard error bars of the mean are indicated. **(C)** FGF2, SDC1, and CD30 mRNA expression levels in normal lymph node controls (NC, solid gray bar) and HL tissues associated with good outcome (GO, striped bar) and poor outcome (PO, solid black bar) were analyzed by qRT-PCR. The measurements represent the fold change after normalization with the NC group. **(D)** The same set of normal and HL tissues from **(B)** were immunostained for FGF2, SDC1, and CD30. Representative normal and stage II GO and PO patients are shown. **(E)** CD20 expression in normal lymph nodes and HL tissues analyzed by immunostaining. The significance of all qRT-PCR data comparing GO and PO is indicated (p<0.005). Scale bars represent 100 μm.

Established HL cell lines potentially represent poor outcome HL because they were generated from primary HRS cells isolated from extranodal sites of pleural effusion, bone marrow, or peripheral blood. Extranodal HL implies lymphatic and hematogenous dissemination via circulation. We screened ten HL cell lines for altered expression of a set of bioinformatics-identified genes representing multiple signaling pathways such as apoptosis, proliferation, angiogenesis, and metastasis (Table 2). The qRT-PCR results revealed that, compared to their expression by primary B cells, FGF2 (Fibroblast Growth Factor 2) and SDC1 (Syndecan1) were overexpressed in eight of the ten cell lines (Figure 2A).

To determine whether FGF2 and SDC1 were overexpressed specifically in HL patient samples, 48 HL and 116 major subtypes of non-Hodgkin lymphoma (NHL) tissue sections in a tissue microarray format were analyzed by immunohistochemical methods (Figure 2B). Qualitative scoring of immunostaining showed that FGF2 and SDC1 were predominantly overexpressed in HL compared to NHL or normal lymph nodes (p < 0.05). To investigate the gene expression profile of FGF2 and SDC1 in HL tissues, 67 archived HL samples with clinical outcome data were analyzed by qRT-PCR and immunohistochemical methods. The PCR data showed that, when compared to normal lymph node controls, all HL tissues overexpressed FGF2 and SDC1, but tissues from poor outcome patients (n=9) showed 246- and 91-fold increases in FGF2 and SDC1 levels, respectively, while tissues from good outcome patients (n=20) had only 10- and 2-fold respective increases. Thus, the poor outcome group expressed 24-fold more FGF2 and 56-fold more SDC1 than the good outcome group (Figure 2C). Expression of CD30 was increased by 59-fold in the poor outcome group and 3-fold in the good outcome group, suggesting that the fold-difference between the poor and good outcome groups is largely contributed by CD30 positive (CD30+) cells in the poor outcome group. Immunostaining of FGF2 and SDC1 was intense in the poor outcome group but weak to moderate in the good outcome group (Figure 2D). In HL tissues from the poor outcome group, CD30^+^FGF2^+^SDC1^+^ cells were seen in clusters in whole mount HL tissues (data not shown). Immunostaining of the same tissues indicated that CD20 expression was significantly reduced in all HL tissues compared to normal controls (Figure 2E), suggesting that the increase in staining and gene expression of FGF2 and SDC1 in the poor outcome group is a consequence of increased numbers of CD30+ cells rather than of CD20+ B-cells.

### CD30+ cells coexpress FGF2 and SDC1 in macrophage-rich tissues from the poor outcome group of HL patients

**Figure 3:**
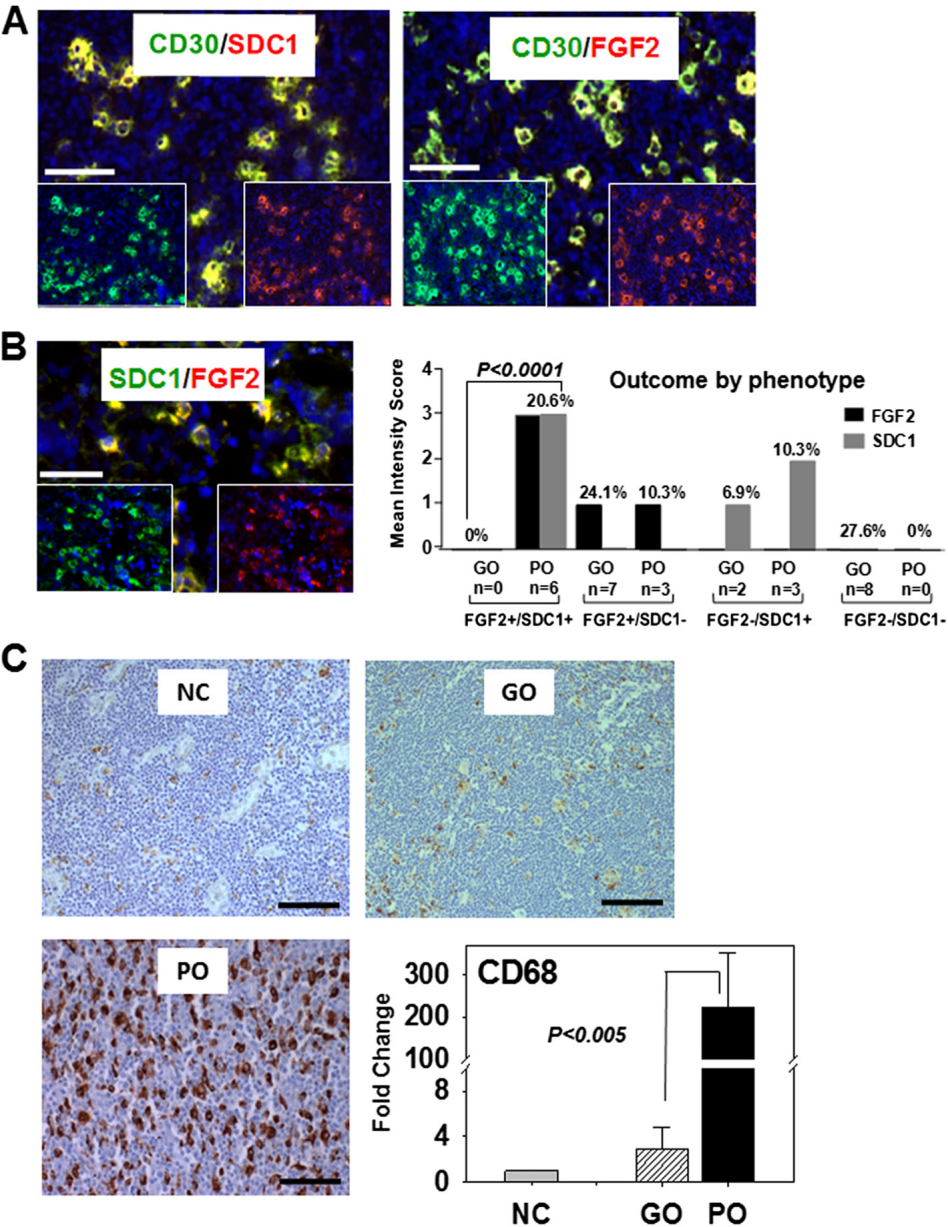
**CD30+ cells coexpress FGF2 and SDC1 in macrophage-rich HL tissues with poor outcome. (A)** Double immunofluorescent staining showing expression of either FGF2 or SDC1 by CD30+ cells of poor outcome samples. Individual green or red fluorescence is depicted at the bottom of each image; scale bar (white solid bar) represents 100 μm. **(B)** Distribution of the immunophenotypes by outcome. The mean intensity scores for FGF2 (solid gray bar) and SDC1 (solid black bar) (y-axis) for the good outcome (GO) and poor outcome (PO) groups of HL patients. Immunofluorescence intensity was scored as 0 (no staining), 1 (weak), 2 (moderate), or 3 (strong) for FGF2+ or FGF2- and SDC1+ or SDC1-. The frequency (%) of expression of each combination of FGF2+/− and SDC1+/− among all tissue sections is indicated above each bar. **(C)** CD68 macrophage marker expression was analyzed by immunostaining (image) and qRT-PCR (graph) in normal lymph node control (NC), good outcome (GO), and poor outcome (PO) groups of HL patients. The fold-change in CD68 mRNA was calculated after normalization with NC. Significance of all qRT-PCR data comparing GO and PO is indicated for **(B)** and **(C)** (p < 0.005). Scale bars represent 100 μm.

Double immunofluorescence analysis of HL tissues showed that all sections from the poor outcome group had clusters of CD30+ cells that coexpressed FGF2 or SDC1 (Figure 3A). The majority of tissues showed weak or no FGF2 or SDC1 staining or weak staining for both FGF2 and SDC1 (Figure 3B graph). All FGF2+/SDC1+ cells with intense fluorescence (n=6) were associated with the poor outcome group, and often clustered in several regions within the whole mount HL tissues. Clusters of FGF2-/SDC1+ and FGF2+/SDC1- cells were seen in each of the remaining poor outcome HL tissues (n=3). Also, clusters of FGF2+/SDC1- or FGF-/SDC1- cells were seen in good outcome HL tissues (Figure 3B-graph). These results suggest that FGF2 and SDC1 coexpression in CD30+ cells or in clusters of cells may trigger molecular signaling that contributes to a poor clinical outcome. A Kaplan-Meier analysis also indicated that the FGF2+/SDC1+ immunophenotype of CD30+ cells is associated with shortened survival (not shown).

CD68+ tumor-associated macrophages were recently shown to be associated with adverse outcomes, including shortened survival [24], which is a consequence of primary refractory and early relapsing cHL. Therefore, we evaluated the number of CD68+ tumor-associated macrophages in the good and poor outcome groups. More CD68+ tumor-associated macrophages were present in the PO group than in either the GO group or among normal controls (Figure 3C). CD68 immunostaining was also more intense in the PO group than in the other groups (Figure 3C). The analysis of CD68+ tumor-associated macrophages and IHC staining data were verified by qRT-PCR, which demonstrated that CD68 expression in the poor outcome group was 77-fold greater than in the good outcome group, and 224-fold greater than in normal lymph nodes (Figure 3C, graph). These increases suggest that a large tumor macrophage population promotes poor clinical outcome by potentiating aggressive CD30+ tumor cells in a subset of HL patients, and some of these CD30+ cells may express FGF2 and SDC1.

### The metastatic markers TGFβ1 and MMP9 are overexpressed in the poor outcome group of HL patients and by HL cell lines

**Figure 4:**
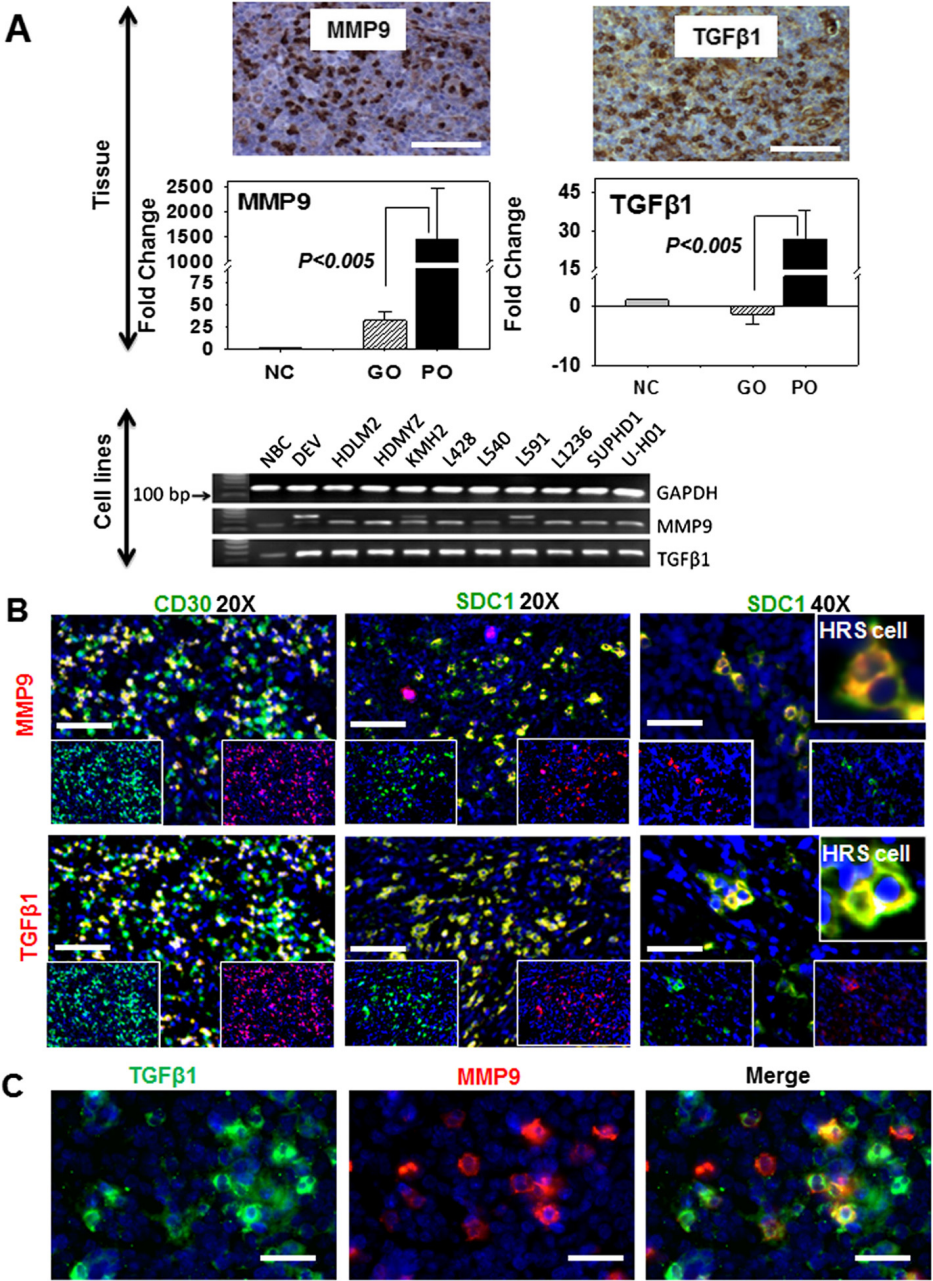
**Metastatic markers TGFβ1 and MMP9 are overexpressed in poor outcome HL patients and by HL cell lines. (A)** Protein and mRNA expression levels of TGFβ1 and MMP9 in normal lymph node control (NC), good outcome (GO) group and poor outcome (PO) group analyzed by immunostaining (left, images only for PO group) and qRT-PCR (right). mRNA expression is represented by fold-change (y-axis) after normalization with the control (NC). Significance of all qRT-PCR data comparing GO and PO is indicated (p < 0.005). TGFβ1 and MMP9 are also overexpressed by the HL cell lines (lower image of gel electrophoresis of **(A)**). **(B)** TGFβ1 and MMP9 protein coexpression in tissues from the poor outcome HL patient group analyzed by double immunofluorescence staining for CD30, TGFβ1 and MMP9, or SDC1, TGFβ1 and MMP9. Individual green or red fluorescence is depicted at the bottom of each image. **(C)** Coexpression of TGFβ1 and MMP9 by subsets of tumor cells in poor outcome sample. (Inset of **A** and **B**) Hodgkin Reed Sternberg cells (HRS) coexpressing SDC1 and TGFβ1 or SDC1 and MMP9. Scale bar (white solid bar) represents 100 μm.

Poor prognosis in HL typically correlates with the presence of tumor cells in extranodal sites distant from the primary tumor. To investigate the metastatic potential of HL tissues having an abundance of CD30+/FGF2+/SDC1+ cells and poor clinical outcome, tissue sections were immunostained for TGFβ1 and MMP9 expression (Figure 4A). The HL tissues from the poor outcome group stained intensely for MMP9 and TGFβ1 compared with the good outcome group and with normal lymph nodes (Figure 4A). Quantitative analysis of MMP9 and TGFβ1 gene expression in the poor outcome group showed increases of 45- and 52-fold, respectively, compared to the good outcome group (after normalization against normal lymph nodes). The mean increase in MMP9 expression in the poor outcome group was 1457-fold while the good outcome group had levels that were increased by 26-fold compared to normal lymph nodes, suggesting that poor outcome HL tissues have high metastatic potential. Because the HL cell lines potentially represent poor outcome, the expression of MMP9 and TGFβ1 was analyzed by PCR. We found that HL cell lines expressed more MMP9 and TGFβ1 than normal B cell (Figure 4A). Double immunofluorescence analysis showed that a subpopulation of CD30+ cells overexpressed TGFβ1 and MMP9 (Figure 4B and 4C), suggesting that CD30+/TGFβ1+ and CD30+/MMP9+ cells may potentiate a metastatic environment that allows CD30+ HL tumor cells to exit the local tumor microenvironment.

### FGF2 and SDC1 are overexpressed in putative circulating CD15+/CD30+ cells in poor outcome HL patients

**Figure 5:**
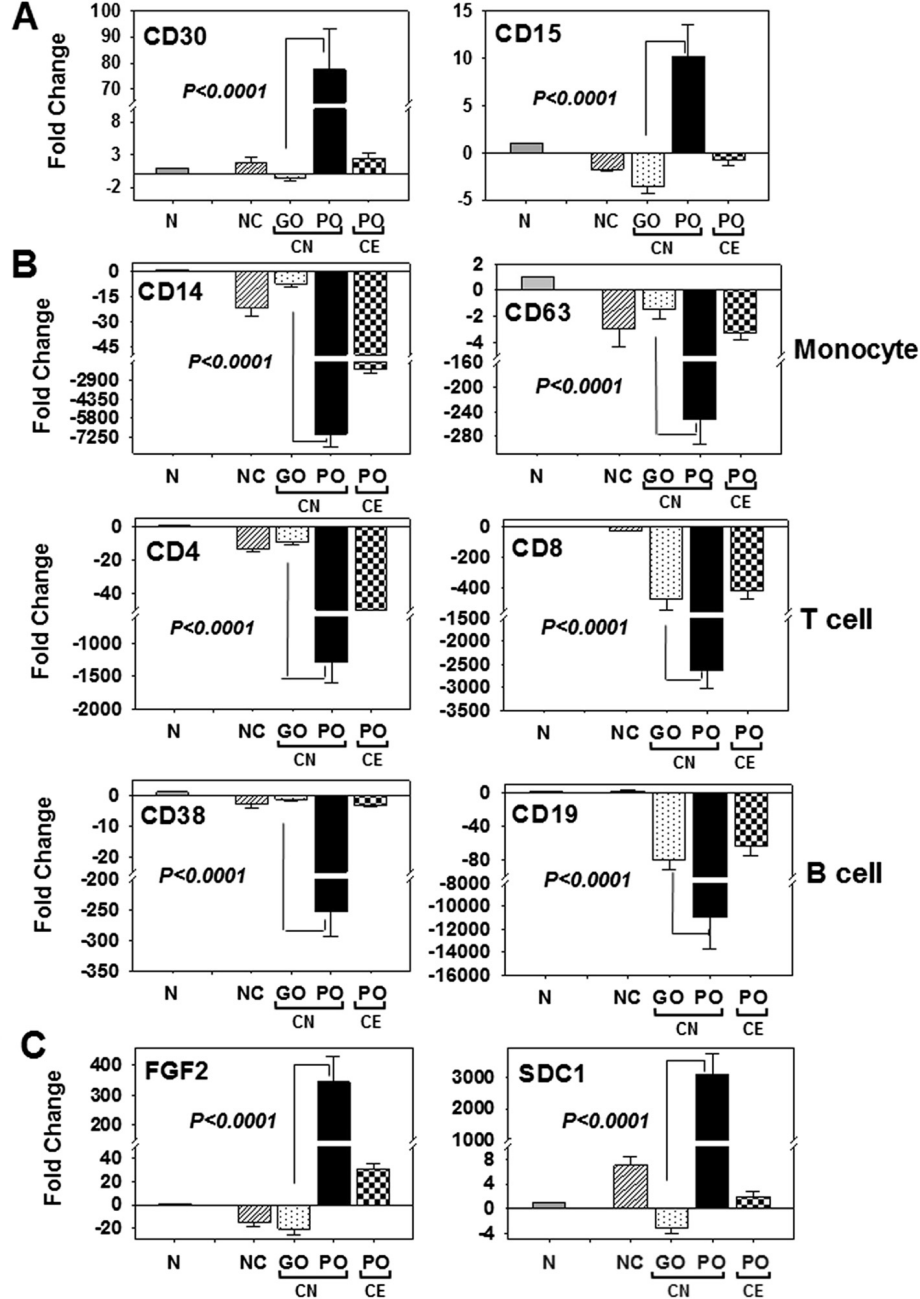
**FGF2 and SDC1 are overexpressed in circulating CD15+/CD30+ cells from chemo-naive poor outcome HL patients.** qRT-PCR analysis of cells isolated from the buffy-coat of peripheral blood from normal donor controls (NC, striped bar), chemo- naïve (CN) good outcome (GO, dotted) and CN poor outcome (PO, solid black bar) groups, and chemo-exposed PO group (CE, checkered bar). Expression levels are represented as fold-change (y-axis) after normalization with normal control cells (N, solid gray bar: N denotes B cells in A and C; N denotes monocytes, CD4 T cells, CD8 T cells, and CD19 B cells in B). **(A)** mRNA expression of CD30 and CD15; **(B)** cell-specific markers for monocytes (CD14, CD63), T-cells (CD4,CD8), and B-cells (CD38, CD19); **(C)** FGF2 and SDC1. Significance of all qRT-PCR data comparing chemo-naïve GO and chemo-naïve PO is indicated (p < 0.0001; ANOVA and PLSD).

To determine whether a subpopulation of CD30+ tumor cells was potentially being shed from the local tumor microenvironment and entering the circulation, we analyzed PBL samples collected from HL patients either prior to frontline treatments (chemo-naive: CN) or after treatment for multiple relapses (chemo-exposed: CE). In baseline HL patients, qRT-PCR results showed that cells from the poor outcome group overexpressed CD15 and CD30 by 41-fold and 113-fold, respectively, compared to the good outcome group after normalization with respect to purified B cells (Figure 5A). In this analysis, the significant increase in marker expression seen for the poor outcome groups was eliminated in the chemo-exposed poor outcome group (Figure 5A), suggesting that CD15+/CD30+ cells in the circulation were killed by chemotherapy treatments. A moderate difference in marker expression between the CN good outcome group and the normal control group (n=10) was observed. To determine if the circulating cells overexpressing CD15+/CD30+ originated from other cell types in the blood, the expression levels of established cell-specific markers, including CD14 (monocytes, macrophages, neutrophils, granulocytes, and dendritic cells), CD63 (basophil activation), CD4 (helper T-cells), CD8 (cytotoxic T cells), CD38 and CD19 (B cells) were analyzed (Figure 5B). Among CN HL patients, compared to the good outcome group, a significant down-regulation of CD14 (−7150-fold), CD63 (−966-fold), CD4 (−1287-fold), CD8 (−2625-fold), CD38 (−253-fold) and CD19 (−10954-fold) expression was seen for the poor outcome group (Figure 5B). In these analyses, the expression levels of CD8, CD38, and CD19 in chemo-exposed HL patients were similar to levels in the good outcome group of CN patients, although the down-regulation of CD8 and CD19 expression was significantly lower (−125-fold for CD8 and −19085-fold for CD19) than that in normal samples. Although the down-regulation of CD14, CD63, CD4, and CD38 among the CN good outcome group of HL patients was similar to normal controls, CD8 and CD19 were significantly down-regulated (CD8 by −125-fold and CD19 by −19085-fold) in the good outcome CN patients compared to normal samples (Figure 5B). The down-regulation signatures of the cell markers in the CN poor outcome group were directly opposite that of the CD15+/CD30+ upregulation signature, suggesting that CD15+/CD30+ cells in the CN poor outcome group were potentially derived from circulating HL tumor cells (Figure 5A and 5B). In these circulating cells, FGF2 and SDC1 genes were overexpressed by 17- and 9764-fold, respectively, compared to the good outcome group (Figure 5C). This fold-difference was reduced in relapsing HL patients in CE group relative to the CN good outcome group, indicating that FGF2 and SDC1 are robust baseline biomarkers for predicting clinical outcomes for CN HL patients.

## Discussion

The survival time for high risk, unfavorable cHL (early relapse, progressive disease, primary refractory) ranges from 0 to less than 4 years [1,25], a very poor prognosis indeed, considering the high cure rate enjoyed by HL patients with current standard therapy. Second line treatments which include ASCT, and chemotherapy plus radiation do not significantly improve the prognosis of this group of patients. Therefore, it is essential to identify biomarkers that can help predict, prior to treatment which cHL patients belong in the high risk group so that appropriate treatment options with potentially better outcome can be implemented. The results presented here suggest that coexpression of FGF2 and SDC1 by CD30+ cells identify this group of patients.

Previous studies showed advanced stage, advanced age, and bulky disease as important risk factors for poor outcome [16]. However, a contingency analysis performed on the HL database from the Tissue Repository of the Hackensack University Medical Center showed no association of any of these risk factors, including treatment history (all p > 0.1) with patient outcome. These data suggest that there may be undetermined molecular pathways that are altered in subsets of NS-cHL patients who are predisposed to be primary refractory or experience multiple relapses shortly after frontline treatments. To improve the specificity of potential biomarkers that may assist in pre-selecting poor outcome patients prior to treatment, we used bioinformatic data mining to derive a list of over 150 genes that represent pathways for metastasis, apoptosis, cell proliferation, tumorigenesis and angiogenesis. Expression screening data for these genes showed a consistent and robust overexpression of FGF2 and SDC1 in HL cell lines that were originally derived from primary HRS cells isolated from extranodal sites of refractory or relapsing HL patients. Qualitative scoring by IHC on lymphoma tissue arrays showed that FGF2 and SDC1 expression were indeed specific to the HL tumor microenvironment. Further analyses by qRT-PCR showed overexpression of either gene in poor outcome samples, while additional IHC on the poor outcome samples showed regions of CD30+ cells where FGF2 and SDC1 were strongly expressed. Double immunofluorescence staining of samples from poor outcome biopsies showed large subsets of CD30+ cells that expressed either FGF2 or SDC1. qRT-PCR and IHC evaluation of CD68 expression confirmed the clinical status of the biospecimens (poor outcome). The metastatic markers MMP9 and TGFβ1 were shown to be overexpressed in poor outcome patient samples, including their overexpression by subsets of CD30+ cells, suggesting metastasis by these HRS cell subsets. qRT-PCR analyses of PBL showed that CD30 and CD15 (the gene which encodes the protein that transfers fucose to N-acetyllactosamine polysaccharides to generate fucosylated carbohydrate structures) were transcriptionally upregulated in the untreated, poor outcome group compared to other clinical groups. Concurrently, markers representing circulating T cells (CD4 and CD8), B cells (CD19 and CD38), and monocytes (CD14 and CD63) were significantly downregulated in the untreated, poor outcome group, indicating that CD30 and CD15 upregulation was not a consequence of their expression by other common circulating lymphocytes. In the untreated, poor outcome group, transcription of FGF2 and SDC1 was upregulated, most likely by CD30+/CD15+ cells. Taken together, these data indicate that in untreated, poor outcome patients, a subset of CD30+ cells that express high levels of FGF2 and SDC1 transcripts, perhaps HRS cells, made their way into the circulation, and may be responsible for the poor outcome generated in primary refractory and early relapsing NS-cHL patients.

The expression of either FGF2 or SDC1 seen in our study is only partially consistent with previous reports. A previous immunoblot analysis showed no expression of FGF2 by HL cell lines KM-H2 and L428, although the same study did detect FGF2 expression in primary HRS cells from HL tumor biopsy samples [26]. In contrast, our qRT-PCR data showed that FGF2 is transcriptionally upregulated in both KM-H2 and L428 cell lines. In HL, it appears as though FGF2 transcript translation in HRS cells is only induced *in vivo*. Although perhaps not completely relevant to HL, elevated FGF2 mRNA is thought to be involved in tumor development and progression, as was demonstrated in acoustic neuromas [27]. Also, there is discordance among studies of SDC1 expression by HRS cells. Studies have reported that the percentage of SDC1-positive HRS cells varies from 0 to 50% among cHL cases [28-31], which is consistent for a post-germinal center origin. These differences may be due to variations in the fixation techniques and SDC1 antibody clones used; for example, some investigators contend that a much higher percentage of SDC-1 positive cells is seen in frozen material compared to formalin fixed paraffin embedded material. Our double immunofluorescence staining on fresh frozen sections from PO group patients showed large subsets of HRS cells that costained with anti-CD30 (clone Ber-H2) and anti-SDC1 (clone BB4 and a polyclonal antibody from Sigma-Aldrich).

The upregulation of FGF2 and SDC1 by putative CD30+ cells observed in our study may be the result of unregulated, uncontrolled expression of these genes in HRS cells from PO patients. Dysregulation of either FGF2 or SDC1 signaling alone or together has been associated with a variety of malignancies, including those associated with poor prognosis. Disruption of FGF2 expression results in elevated serum FGF2 levels, which is an independent poor prognostic factor for lymphoma, lung cancer, and sarcoma patients [32-35]. In addition, elevated levels of FGF2 in serum have been reported for non-Hodgkin lymphoma (NHL) patients with poor prognosis [36], shortened survival, and higher risk for mortality [35]. Also in lymphoma, FGF2 overexpression in diseased tissue biopsy samples is associated with chemoresistance and inferior progression free and overall survival [37]. Kowalska et al. (2007) showed that elevated FGF2 serum levels correlated with the erythrocyte sedimentation rate, which is a poor prognostic factor in HL [16,38]. At a molecular level, FGF2 binds to multiple membrane bound receptors in human cancers, including SDC1 [39], and this receptor binding can trigger multiple signaling pathways, including those involved in cell proliferation and survival [40,41]. Although FGF2 may not always mediate SDC1 expression in cancers, SDC1 overexpression, at either a tissue or serum level, has been reported for multiple tumor types including solid tumors [42-44], lymphomas, and in a number of lymphoproliferative disorders [29,30,45-47]. In some instances, SDC1 overexpression is an adverse indicator for both solid and hematological malignancies [43,44,48-50]. High levels of FGF2 and SDC1 in the same patient have important clinical implications. Multiple myeloma patients and small cell lung cancer patients with high serum levels of soluble SDC1 and FGF2 have poor prognosis and shortened survival [51]; high serum levels of soluble SDC1 and FGF2 are also important clinical features of high risk, primary refractory, early relapsing cHL, and untreated poor outcome patients in our study. However, the clinical significance of co-upregulation of SDC1 and FGF2 in serum of HL patients has yet to be explored.

Our results also revealed that a large number of CD68+ tumor-associated macrophages were present in the tumor microenvironment of poor outcome tissue samples in which the CD30+ cells overexpressed both SDC1 and FGF2. A number of previous reports showed that CD68+ tumor-associated macrophages are a poor outcome marker of cHL [52-54]. Therefore, simultaneous overexpression of FGF2 and SDC1 by CD30+ cells can be used as a molecular signature to identify high risk, poor outcome cHL patients.

The downregulation of markers representing circulating T cells, B cells and monocytes in the PBL of untreated poor outcome patients in our study is consistent with lymphocytopenia, a negative prognostic factor in multiple cancer types [55]. However, lymphocytopenia alone may not adequately predict poor prognosis, and as such better biomarkers are needed. Two recent reports indicated that the ratio of the absolute lymphocyte count to the absolute monocyte count (ALC/AMC) is an independent prognostic marker that can be used for stratifying high vs. low risk cHL [56-58]. Although not demonstrated in hematological malignancies, lymphocytopenia implies a depleted immune system that lacks adequate immune surveillance, which could play an important role in aggressive tumor metastasis. Indeed, both lymphocytopenia and circulating tumor cells were shown to be independent prognostic factors in the metastasis of breast cancer, carcinomas, sarcomas, and lymphomas; their presence is associated with an extremely poor clinical outcome [55,59]. Such a scenario may play a role in extranodal involvement of HL, and may identify unfavorable high risk patients irrespective of disease stage. In such a setting, this population of HL patients may benefit from therapies to restore immune function prior to frontline therapy.

In some ways, the co-upregulation of FGF2 and SDC1 seen in tissue biopsies and PBL (albeit at the mRNA level) of PO NS-cHL patients in our study may be related to certain features of multiple myeloma. Several studies found shared features between HRS cells and plasma cells, including those of multiple myeloma and their normal counterparts, despite the differences in disease behavior [30,60-62]. Like plasma cells, HRS cells typically not only lack expression of B-cell surface markers, but they are also the only other lymphocytes that occasionally express SDC1 [30,60-63]. Although B lymphocyte–induced maturation protein 1 (Blimp-1), which is a transcription factor required for plasma cell differentiation [62], was not part of our study, a fraction of HRS cells also express this protein. There is the possibility that subsets of, if not all, HRS cells and multiple myeloma plasma cells share a common ancestral precursor [64], although the majority of HRS cells shed their plasma cell signature (e.g., SDC1 expression) [62]. Those HRS cells that continue to express SDC1 are perhaps more aggressive than their SDC1 negative counterparts, thus contributing to the aggressive nature of poor outcome cHL, and producing the sort of unfavorable prognosis that is typical of primary refractory and early relapsing cHL patients. Of additional interest is the coexistence of HRS cells with aggressive multiple myeloma [65], or their appearance after treatment of multiple myeloma [66].

Our data also showed that the established metastatic markers MMP9 and TGFβ1 were overexpressed by subsets of CD30+/FGF2+/SDC1+ cells in tissue biopsy samples from PO patients. HRS cells produce activated TGFβ1 in primary tumor tissues, predominantly in nodular sclerosing HL [67], while MMP9 overexpression is associated with adverse clinical outcomes in HL [68]. As such, HRS cells that harbor the FGF2+/SDC1+ immunophenotype and express both MMP9 and TGFβ1 are the cells most likely to be shed from the tumor microenvironment. Thus, the molecular interplay of FGF2, SDC1, MMP9, and TGFβ1 may play a role in HL metastasis.

Finally, our results revealed that subsets of circulating cells transcriptionally upregulate CD30, CD15, FGF2, and SDC1 in the untreated poor outcome group. The main subset here could be putative HRS cells or some other variant of these neoplastic cells that are SDC1+/FGF2+ and overexpress MMP9 and TGFβ1. While characteristic HRS cells are not typically found in PBL, the metastatic and hematogenous spread of HL is suspected in cases diagnosed with extralymphatic and extranodal involvement [15]. Therefore, a variant of HRS cells that do not exhibit the classical phenotype displayed by nodal HRS may be in the circulation of untreated poor outcome patients, perhaps due to a difference in the microenvironment (PBL versus lymph node). In other settings, either normal cells or circulating tumor cells may express important transcripts that are translated only when the appropriate microenvironment prevails, and thus the cell phenotype may also change. This concept is evident during development during which the zygote produces maternal RNAs that are later translated into functional proteins at each stage during embryogenesis. At least two studies of HL patient subsets suggest a similar occurrence. In vitro experiments by Zucker-Franklin and colleagues (1983) and Sitar et al. (1994) showed that RS-like cells can be generated from cultured peripheral mononuclear blood cells (PMBC) from HL patients [69,70]. Zucker-Franklin et al. observed RS-like cells only in HL samples (including early stage disease), and not PMBC of NHL, mycosis fungoides, or of control samples, suggesting that giant cell formation from PMBC is limited to HL cases. Sitar and colleagues showed that 10% of the giant RS-like cells were CD30+ and EBV-positive [70].

## Conclusions

Our study used bioinformatics analysis to identify biomarkers that could be helpful in identifying HL patients who are predisposed to a poor outcome, and could be helpful in directing these patients to the optimal treatment regimen. In poor prognosis HL patients, we found small subsets of circulating CD30+/CD15+ cells that express FGF2 and SDC1; these proteins may be appropriate biomarkers for HL prognosis.

## Methods and materials

### Bioinformatics

The BioXM software platform (Sophic Alliance, Rockville, MD) was used to mine potential biomarkers for Hodgkin’s lymphoma using the National Cancer Institute (NCI) Cancer Gene Index, which contains 7,000 cancer genes and 2,200 biomarker genes. These genes were annotated and validated from 18 million Medline abstracts and 24,000 Hugo genes from over 80 databases, using a combination of algorithmic methods (Biomax Informatics, Munich, Germany) that included natural language processing (NLP), Biomarker Role Codes, the NCI Cancer Thesaurus, and Karp’s Evidence Codes [23]. The identification of potential biomarkers was performed by initiating queries on BioXM with a combination of search terms including Hodgkin’s disease, lymphoma, cancer, biomarker, overexpression, up-regulation or down-regulation, and differentially-expressed. The bioinformatics-guided search generated 151 potential HL biomarkers (Table 2).

### Cell lines and cell culture

The Hodgkin’s lymphoma cell lines KM-H2, HD-MY-Z, HDLM-2, L-591, and SUP-HD1 were obtained from the German Collection of Microorganisms and Cell Cultures (Braunschweig, Germany). L-428, L-1236, and L-540 cells were generous gifts provided by Dr. Volker Diehl (University of Cologne, Germany). U-H01 and DEV cells were kind gifts from Dr. S. Brüderlein (University Hospital Ulm, Germany) and Dr. Debora De Jong (Netherlands), respectively. KM-H2, L-428, HD-MY-Z, and L-1236 cells were cultured in 90% RPMI 1640 supplemented with 10% fetal bovine serum (FBS). SUP-HD1 cells were grown in 80% McCoy’s 5A medium containing 20% FBS. HDLM-2, L-540, and L-591 cells were grown in 80% RPMI 1640 supplemented with 20% FBS. U-H01 cells were grown in Iscove’s MDM and RPMI 1640 (4:1) supplemented with 20% FBS. All culture media contained 2 mM L-glutamine, penicillin (100 U/ml), and streptomycin (0.1 mg/ml). Cultures were maintained at 37°C with 5% CO_2_. The clinical characteristics of each cell line were previously documented and are presented in Table 3. DEV, KM-H2, and SUP-HD1 cells were derived from relapsing cases. HD-MY-Z, L1236, L428, and U-H01 cells were from refractory patients.

**Table 3:** Characteristics of HL cell lines

**Cell line**	**Clinical characteristic**	**Anatomic site of primary cell**
DEV	relapse	Pleural fluid
HDLM2	n/a	Pleural fluid
HD-MY-Z	refractory	Bone marrow
KM-H2	relapse	Pleural fluid
L1236	refractory/relapse	Peripheral blood
L428	refractory	Pleural fluid
L540	n/a	Bone marrow
L591	n/a	Pleural fluid
SUP-HD1	relapse	Pleural fluid
U-H01	refractory	Pleural fluid

A review of the literature showed that all established HL cell lines were derived from primary malignant CD30+ cells isolated from extra-nodal sites: pleural fluid, bone marrow, and peripheral blood. No cell lines to date have been raised from primary HRS cells isolated from lymph nodes.

### RNA isolation and cDNA synthesis

Total RNA from cell lines and peripheral blood (PBL) of HL patients was isolated using Trizol (Invitrogen, Carlsbad, CA). RNA from archived FFPE tissue sections was extracted using RNeasy (Qiagen, CA) according to the manufacturer’s instructions. The RNA concentration was spectrophotometrically determined at A260 (ThermoElectro Corporation). Total RNA integrity was checked by resolution on a 2% agarose gel under denaturing conditions. cDNA was generated using the SuperScript III RT First-Strand cDNA Synthesis Kit (Invitrogen, Carlsbad, CA) according to the manufacturer’s protocol. Oligo-dT primers were used to generate cDNA from cell lines and PBL-derived RNA, and random hexamers were used for generating cDNA from RNA obtained from FFPE sections.

### Polymerase chain reaction (PCR)

Primer sets used for each gene were generated using online primer tools (University of Massachusetts; http://biotools.umassmed.edu/bioapps/primer3_www.cgi) (Table 2). Primers were designed to have lengths of 18 to 27 nt with Tm = 60°C and 45 to 65% GC content, and were synthesized by a custom primer service provided by Invitrogen. Each primer pair was confirmed to generate a single discrete band by end-point PCR (BioRad DNA Engine Peltier Thermal Cycler) using cDNAs generated from normal spleen tissue. End-point PCR conditions consisted of denaturation at 95°C for 30 seconds, annealing at 55°C for 30 seconds, and primer extension at 72°C for 1 minute. The primer pairs were designed to generate a PCR fragment of 150–170 bp for cell line- and PBL-derived cDNA, and 70–100 bp for FFPE-derived cDNA (Table 4). The PCR products were resolved on a 2% agarose gel and visualized with ethidium bromide staining using a BioRad Imager. For qRT-PCR, each reaction consisted of 43 ng cDNA, 10 mmole primers and 10 μl 2X Power SYBR Green PCR Master Mix (Applied Biosystems, Foster City, CA) in a final volume of 20 μl, which was placed in a MicroAmp Fast Optical 96-Well Reaction Plate designed for use with the ABI7900 PCR system (Applied Biosystems). The reaction was performed using the standard mode (initial denaturation at 95°C for 10 minutes followed by 40 cycles of 95°C for 15 seconds and 60°C for 1 minute). Each qRT-PCR reaction was done in triplicate, and each data set was analyzed with ABI7900 software. The amount of target mRNA was normalized to the expression levels of the housekeeping gene GAPDH. For cell lines, CD19 was used as control. For PBL analysis, the expression levels of CD14/63, CD38/19, and CD4/8 were compared against their expression in monocytes, CD19+ B cells, helper T cells, and cytotoxic T cells, respectively, of healthy donors (Miltenyi Biotech). Pooled normal cDNA (n=20) was used as a control for gene expression analysis of FFPE tissue-derived cDNA. The ΔΔCt method was used to calculate the fold-change relative to controls.

**Table 4:** Primer sets for each gene used in this study

***Genes***	***Forward sequence***	***Reverse sequence***
A. Primer sets used on PBL samples.		
GAPDH	catggcctccaaggagtaag	aggggtctacatggcaactg
CD4	atgtggcagtgtctgctgag	cctagcccaatgaaaagcag
CD8	cagagctacccgcagagttc	ctccaaccctgacttgctgt
CD30	ccaacttagctgtcccctga	ctgggaccaatgctgttctc
CD15	gcaggtgggactttgttgtt	ccaaggacaatccagcactt
CD19	ttctgcctgtgttcccttg	cacgttcccgtactggttct
CD38	agatctgagccagtcgctgt	aaaaaggcttccgtctctgg
CD14	gagctcagaggttcggaaga	ttcggagaagttgcagacg
CD63	aaccacactgcttcgatcct	aatcccacagcccacagtaa
FGF2	tgctcagcagtcaccatagc	cttgaggtggaagggtctcc
SDC1	cttcacactccccacacaga	ggccactacagccgtattct
B. Primer sets used on FFPE tissues.		
GAPDH	cctcaacgaccactttgtca	ccctgttgctgtagccaaat
TGFβ	gtacctgaacccgtgttgct	cacgtgctgctccactttta
MMP9	ggcgctcatgtaccctatgt	gccattcacgtcgtccttat
CD30	gaagctccacctgtgctacc	ggtctggaatccacaagctc
CD68	tgacacccacggttacagag	gtggttttgtggctcttggt
SDC1	taggacctttccaccacagc	gaggctgcttcagtttggag
FGF2	tgaggctgagaggtcaaggt	ctctgttgcctaggctggac

### Selection of clinical samples

The selection criteria of peripheral blood samples were based on the response to front line therapy (Table 1). Twenty five nodular sclerosing cHL patient samples registered in the database at the Hackensack University Medical Center were categorized into: 1) good outcome chemo-naïve, untreated, relapse-free/disease-free > 4 years (n=12); 2) poor outcome chemo-naïve (untreated), primary refractory or early relapse (n=7); 3) chemo-exposed (pretreated), multiple relapses (n=6). Formalin-fixed, paraffin-embedded (FFPE), and fresh frozen (FF) lymph nodes from different HL stages and subtypes were obtained from Thomas Jefferson University, the Tissue Repository of the Hackensack University Medical Center, and Proteogenex (Culver City, CA). Biospecimens with the relevant clinical characteristics were grouped into good outcome (GO, relapse free/disease free > 4 years, n=20) and poor outcome (PO, shortened survival— death 2 to 3 years after diagnosis). A lymphoma tissue array was obtained from US Biomax (Rockville, MD).

### Immunohistochemistry

FFPE and fresh frozen lymph nodes from different stages and subtypes of HL were purchased from US Biomax and Proteogenex. FFPE sections (5 μm) mounted on slides were dewaxed twice with Histochoice clearing agent (Amresco, Solon, OH) for 10 minutes each, then sequentially hydrated in 100%, 90%, 80%, 70%, and 50% ethanol followed by equilibration in PBS for 5 minutes each. All antigen retrievals were carried out in a 95°C water bath for 20–30 minutes (depending on the antigen) using high pH (pH 9) buffer (DAKO) for FGF2, SDC1, MMP9, and CD68, or low pH (pH 6) buffer (DAKO) for CD30, TGFβ1, and CD20. The sections were cooled for 20 minutes at room temperature and then washed twice with PBS for 5 minutes. Endogenous peroxidases were quenched by incubating the sections in 3% H_2_O_2_ solution in PBS for 10 minutes followed by rapid washes in PBS at room temperature. A hydrophobic PAP pen (Vector Labs, Burlingame, CA) was used to make a dam around the sections, which were then blocked at room temperature for 2 hours with 1% BSA containing 5% swine serum in PBS, followed by overnight incubation with primary antibodies at 4°C. Monoclonal antibodies for CD30 (clone Ber-H2, DAKO), SDC1 (clone BB4, Abd Serotec), CD68 (clone PG-M1, DAKO), and CD20 (clone L26, DAKO) were used at dilutions of 1:20, 1:40, 1:50, and 1:100, respectively. Rabbit polyclonal antibodies for FGF2 (Santa Cruz), TGFβ1 (Santa Cruz), and MMP9 (DAKO) were used at dilutions of 1:200, 1:200, and 1:100, respectively. Stained sections were washed three times in PBS/0.1% Tween-20 for 5 minutes each and then once in PBS for 5 minutes. Signal detection was carried out using an LSAB kit according to the manufacturer’s instructions (DAKO), with minor modifications. Briefly, sections were incubated in Biotinylated Link for 30 minutes at room temperature and washed three times in 0.1% PBST for 5 minutes each. Sections were then incubated in streptavidin-HRP for 30 minutes and washed as described above. Signals were visualized by incubating the slides in a solution of 1 ml substrate buffer with 1 drop chromogen, and immediately rinsed in tap water. The sections were counterstained with hematoxylin (Vector Labs) for 22 seconds and immediately washed in tap water before mounting with Aqua Mount (Vector Labs). Photomicrographs of stained tissues were generated with an Axio Cam MRc camera coupled to an Axio Imager Microscope (Carl Zeiss, Thornwood, NY). Positive control slides included tonsil for CD20, CD68, and SDC1, and ALCL for CD30 (on lymphoma array). For qualitative scoring, no staining was assigned a score of 0, weak staining 1, moderate staining 2, and intense staining, 3.

### Immunofluorescence

Double immunofluorescence analysis was performed on 5 μm FFPE and OCT-embedded 8 μm fresh frozen (FF) tissue sections that were mounted on positively-charged frosted slides (Histoserv, Germantown, MD). FFPE sections were processed similarly to the preparation used for IHC. OCT-embedded FF sections were thawed at room temperature for 20 minutes, rinsed briefly in PBS, and then fixed in 3.7% formaldehyde (Electron Microscopy Sciences, PA) for 20 minutes at room temperature. The remaining steps for immunofluorescence signal detection were carried out using a TSA Detection system (Invitrogen) according to the manufacturer’s instructions. Monoclonal and polyclonal signals were detected with Alexa Fluor 488 and Alexa Fluor 546, respectively. The antibodies used were the same as for IHC, except that a SDC1 rabbit polyclonal antibody (Sigma-Aldrich) was used for CD30-SDC1 double staining. Slides were counterstained with Hoechst 33342, visualized with a Leica DMI 6000B inverted microscope, and analyzed using Leica MM AF software, version 1.5 (Leica Microsystems). Slides were independently reviewed and verified by two pathologists.

### Statistics

Data analyses were performed using SAS 9.1.3, StatView 5, or JMP 4. Contingency and likelihood ratio analyses were used to determine the independence of staging and prognosis. The mean fold-change for each sample was determined from triplicates of the qRT-PCR data. Analysis of variance (ANOVA) and F statistics were used to determine differences between the means of the poor outcome group and other outcome groups. Fisher’s protected least significant difference (PLSD) was used to determine pair-wise significant differences between group means.

## Keypoints

FGF2 and SDC1 overexpression by circulating CD15+/CD 30+ cells is associated with poor outcome in Hodgkin Lymphoma.

## Abbreviations

ABVD: Adriamycin bleomycin, vinblastine, dacarbazine acc., accelerated; BCPAT: BEAM conditioning pre-auto transplant; CR: Complete remission; CE: Chemo-exposed; CN: Chemo-naive; BEACOPP: Bleomycin etoposide, adriamycin, cyclophosphamide, vincristine, procarbazine, prednisone; CPPV: Chlorambucil procarbazine, prednisone, Vinblastine; DICE: Dexamethasone ifosfamide, cisplatin, etoposide; EPOCH: Etoposide vincristine, and doxorubicin with bolus cyclophosphamide; ESHAP: Etoposide methylprednisolone, Ara-C, and cisplatin; FMPAL: Fludarabine melphalan, pre-allo transplant; GVD: Gemcitabine vinorelbine, liposomal vincristine; HCVAD: Hyper cyclophosphamide vincristine, adriamycin, and dexamethasone; ICE: Ifosfamide carboplatin, etoposide; IGEV: Ifosfamide gemcitabine, and vinorelbine; PT: Post transplant; Rel: Relapse; Ref: Refractory; R: Rituximab; Rad: Radiation; SK: Sloan kettering; Std: Standard; WU: Washington University.

## Competing interests

The authors have no conflict of interest to declare.

## Authors’ contributions

RG and KSS designed the study and wrote the manuscript. RG, JP, CK, SS, NH, and SV performed the experiments; TT and MT provided clinical samples and performed experiments; KN provided bioinformatics software and analyses; RG, AG, PB and KSS analyzed the data; AG and AP reviewed the paper and provided advice. All authors read and approved the final manuscript.

## References

[B1] Josting A Rueffer U Franklin J Sieber M Diehl V Engert A Prognostic factors and treatment outcome in primary progressive Hodgkin lymphoma: a report from the German Hodgkin lymphoma study group Blood 2000 96 4 1280 1286 10942369

[B2] Majhail NS Ness KK Burns LJ Sun CL Carter A Francisco L Forman SJ Bhatia S Baker KS Late effects in survivors of Hodgkin and non-Hodgkin lymphoma treated with autologous hematopoietic cell transplantation: a report from the bone marrow transplant survivor study Biol Blood Marrow Transplant 2007 13 10 1153 1159 10.1016/j.bbmt.2007.06.003 17889351 PMC2083636

[B3] Hasenclever D Diehl V A prognostic score for advanced Hodgkin’s disease. International prognostic factors project on advanced Hodgkin’s disease N Engl J Med 1998 339 21 1506 1514 10.1056/NEJM199811193392104 9819449

[B4] Steidl C Farinha P Gascoyne RD Macrophages predict treatment outcome in Hodgkin’s lymphoma Haematologica 2011 96 2 186 189 10.3324/haematol.2010.033316 21282720 PMC3031684

[B5] Azambuja D Natkunam Y Biasoli I Lossos IS Anderson MW Morais JC Spector N Lack of association of tumor-associated macrophages with clinical outcome in patients with classical Hodgkin’s lymphoma Ann Oncol J Eur Soc Med Oncol/ESMO 2012 23 3 736 742 10.1093/annonc/mdr157 PMC3331732 21602260

[B6] Younes A Bartlett NL Leonard JP Kennedy DA Lynch CM Sievers EL Forero-Torres A Brentuximab vedotin (SGN-35) for relapsed CD30-positive lymphomas N Engl J Med 2010 363 19 1812 1821 10.1056/NEJMoa1002965 21047225

[B7] Tamaru J Hummel M Mikata A Stein H [Hodgkin’s disease: the origin of HRS (Hodgkin and Reed-Sternberg) cells] [Rinsho ketsueki] Japan J Clin Hematol 1996 37 5 426 429 8691589

[B8] Seitz V Hummel M Marafioti T Anagnostopoulos I Assaf C Stein H Detection of clonal T-cell receptor gamma-chain gene rearrangements in reed-Sternberg cells of classic Hodgkin disease Blood 2000 95 10 3020 3024 10807764

[B9] Kuppers R Identifying the precursors of Hodgkin and reed-Sternberg cells in Hodgkin’s disease: role of the germinal center in B-cell lymphomagenesis J Acquir Immune Defic Syndr 1999 21 Suppl 1 S74 S79 10430222

[B10] Steidl C Connors JM Gascoyne RD Molecular pathogenesis of Hodgkin’s lymphoma: increasing evidence of the importance of the microenvironment J Clin Oncol 2011 29 14 1812 1826 10.1200/JCO.2010.32.8401 21483001

[B11] Schreck S Friebel D Buettner M Distel L Grabenbauer G Young LS Niedobitek G Prognostic impact of tumour-infiltrating Th2 and regulatory T cells in classical Hodgkin lymphoma Hematol Oncol 2009 27 1 31 39 10.1002/hon.878 18924115

[B12] Skinnider BF Mak TW The role of cytokines in classical Hodgkin lymphoma Blood 2002 99 12 4283 4297 10.1182/blood-2002-01-0099 12036854

[B13] Aldinucci D Lorenzon D Cattaruzza L Pinto A Gloghini A Carbone A Colombatti A Expression of CCR5 receptors on Reed-Sternberg cells and Hodgkin lymphoma cell lines: involvement of CCL5/Rantes in tumor cell growth and microenvironmental interactions Int J Cancer J Int Du Cancer 2008 122 4 769 776 10.1002/ijc.23119 17935139

[B14] Niens M Visser L Nolte IM van der Steege G Diepstra A Cordano P Jarrett RF Te Meerman GJ Poppema S van den Berg A Serum chemokine levels in Hodgkin lymphoma patients: highly increased levels of CCL17 and CCL22 Br J Haematol 2008 140 5 527 536 10.1111/j.1365-2141.2007.06964.x 18275430

[B15] Guermazi A Brice P de Kerviler EE Ferme C Hennequin C Meignin V Frija J Extranodal Hodgkin disease: spectrum of disease Radiographics Rev Public Radiol Soc N Am Inc 2001 21 1 161 179 10.1148/radiographics.21.1.g01ja02161 11158651

[B16] Noordijk EM Carde P Dupouy N Hagenbeek A Krol AD Kluin-Nelemans JC Tirelli U Monconduit M Thomas J Eghbali H Combined-modality therapy for clinical stage I or II Hodgkin’s lymphoma: long-term results of the European organisation for research and treatment of cancer H7 randomized controlled trials J Clin Oncol J Am Soc Clin Oncol 2006 24 19 3128 3135 10.1200/JCO.2005.05.2746 16754934

[B17] Wulfing P Borchard J Buerger H Heidl S Zanker KS Kiesel L Brandt B HER2-positive circulating tumor cells indicate poor clinical outcome in stage I to III breast cancer patients Cli Cancer Res J Am Assoc Cancer Res 2006 12 6 1715 1720 10.1158/1078-0432.CCR-05-2087 16551854

[B18] Hoshimoto S Shingai T Morton DL Kuo C Faries MB Chong K Elashoff D Wang HJ Elashoff RM Hoon DS Association between circulating tumor cells and prognosis in patients with stage III melanoma with sentinel lymph node metastasis in a phase III international multicenter trial J Clin Oncol J Am Soc Clin Oncol 2012 30 31 3819 3826 10.1200/JCO.2011.40.0887 PMC3478576 23008288

[B19] Wolf J Kapp U Bohlen H Kornacker M Schoch C Stahl B Mucke S von Kalle C Fonatsch C Schaefer HE Peripheral blood mononuclear cells of a patient with advanced Hodgkin’s lymphoma give rise to permanently growing Hodgkin-Reed Sternberg cells Blood 1996 87 8 3418 3428 8605360

[B20] Diehl V Kirchner HH Schaadt M Fonatsch C Stein H Gerdes J Boie C Hodgkin’s disease: establishment and characterization of four in vitro cell lies J Cancer Res Clin Oncol 1981 101 1 111 124 10.1007/BF00405072 7276066 PMC12253201

[B21] Mader A Bruderlein S Wegener S Melzner I Popov S Muller-Hermelink HK Barth TF Viardot A Moller P U-HO1, a new cell line derived from a primary refractory classical Hodgkin lymphoma Cytogenet Genome Res 2007 119 3–4 204 210 18253030 10.1159/000112062

[B22] Hsu SM Zhao X Chakraborty S Liu YF Whang-Peng J Lok MS Fukuhara S Reed-Sternberg cells in Hodgkin’s cell lines HDLM, L-428, and KM-H2 are not actively replicating: lack of bromodeoxyuridine uptake by multinuclear cells in culture Blood 1988 71 5 1382 1389 3359046

[B23] Karp PD Paley S Krieger CJ Zhang P An evidence ontology for use in pathway/genome databases Pacif Sympo Biocomp 2004 190 201 10.1142/9789812704856_0019 14992503

[B24] Steidl C Lee T Shah SP Farinha P Han G Nayar T Delaney A Jones SJ Iqbal J Weisenburger DD Tumor-associated macrophages and survival in classic Hodgkin’s lymphoma N Engl J Med 2010 362 10 875 885 10.1056/NEJMoa0905680 20220182 PMC2897174

[B25] Josting A Wolf J Diehl V Hodgkin disease: prognostic factors and treatment strategies Curr Opin Oncol 2000 12 5 403 411 10.1097/00001622-200009000-00004 10975546

[B26] Khnykin D Troen G Berner JM Delabie J The expression of fibroblast growth factors and their receptors in Hodgkin’s lymphoma J Pathol 2006 208 3 431 438 10.1002/path.1900 16353171

[B27] Murphy PR Myal Y Sato Y Sato R West M Friesen HG Elevated expression of basic fibroblast growth factor messenger ribonucleic acid in acoustic neuromas Mol Endocrinol 1989 3 2 225 231 10.1210/mend-3-2-225 2710130

[B28] Tzankov A Zimpfer A Pehrs AC Lugli A Went P Maurer R Pileri S Dirnhofer S Expression of B-cell markers in classical hodgkin lymphoma: a tissue microarray analysis of 330 cases Modern Pathol J US Can Acad Pathol Inc 2003 16 11 1141 1147 10.1097/01.MP.0000093627.51090.3F 14614054

[B29] O’Connell FP Pinkus JL Pinkus GS CD138 (syndecan-1), a plasma cell marker immunohistochemical profile in hematopoietic and nonhematopoietic neoplasms Am J Clin Pathol 2004 121 2 254 263 10.1309/617DWB5GNFWXHW4L 14983940

[B30] Carbone A Gloghini A Gattei V Degan M Improta S Aldinucci D Canzonieri V Perin T Volpe R Gaidano G Reed-Sternberg cells of classical Hodgkin’s disease react with the plasma cell-specific monoclonal antibody B-B4 and express human syndecan-1 Blood 1997 89 10 3787 3794 9160685

[B31] Costes V Magen V Legouffe E Durand L Baldet P Rossi JF Klein B Brochier J The Mi15 monoclonal antibody (anti-syndecan-1) is a reliable marker for quantifying plasma cells in paraffin-embedded bone marrow biopsy specimens Hum Pathol 1999 30 12 1405 1411 10.1016/S0046-8177(99)90160-0 10667416

[B32] Ruotsalainen T Joensuu H Mattson K Salven P High pretreatment serum concentration of basic fibroblast growth factor is a predictor of poor prognosis in small cell lung cancer Cancer Epidemiol Biomark Prev Pub Am Assoc Cancer Res Cosponsored Am Soc Prev Oncol 2002 11 11 1492 1495 12433733

[B33] Ueno K Inoue Y Kawaguchi T Hosoe S Kawahara M Increased serum levels of basic fibroblast growth factor in lung cancer patients: relevance to response of therapy and prognosis Lung Cancer 2001 31 2–3 213 219 11165400 10.1016/s0169-5002(00)00187-2

[B34] Salven P Teerenhovi L Joensuu H A high pretreatment serum basic fibroblast growth factor concentration is an independent predictor of poor prognosis in non-Hodgkin’s lymphoma Blood 1999 94 10 3334 3339 10552942

[B35] Salven P Orpana A Teerenhovi L Joensuu H Simultaneous elevation in the serum concentrations of the angiogenic growth factors VEGF and bFGF is an independent predictor of poor prognosis in non-Hodgkin lymphoma: a single-institution study of 200 patients Blood 2000 96 12 3712 3718 11090051

[B36] Bertolini F Paolucci M Peccatori F Cinieri S Agazzi A Ferrucci PF Cocorocchio E Goldhirsch A Martinelli G Angiogenic growth factors and endostatin in non-Hodgkin’s lymphoma Br J Haematol 1999 106 2 504 509 10.1046/j.1365-2141.1999.01547.x 10460612

[B37] Pazgal I Zimra Y Tzabar C Okon E Rabizadeh E Shaklai M Bairey O Expression of basic fibroblast growth factor is associated with poor outcome in non-Hodgkin’s lymphoma Br J Cancer 2002 86 11 1770 1775 10.1038/sj.bjc.6600330 12087465 PMC2375415

[B38] Kowalska M Janina K Małgorzata F Beata K Alicja S Joanna T Jan W Serum VEGF and bFGF levels in patients with Hodgkin’s lymphoma NOWOTWORY J Oncol 2007 57 4 179e 182e

[B39] Salmivirta M Heino J Jalkanen M Basic fibroblast growth factor-syndecan complex at cell surface or immobilized to matrix promotes cell growth J Biol Chem 1992 267 25 17606 17610 1517210

[B40] Su G Blaine SA Qiao D Friedl A Shedding of syndecan-1 by stromal fibroblasts stimulates human breast cancer cell proliferation via FGF2 activation J Biol Chem 2007 282 20 14906 14915 10.1074/jbc.M611739200 17344212

[B41] Fitzgerald ML Wang Z Park PW Murphy G Bernfield M Shedding of syndecan-1 and −4 ectodomains is regulated by multiple signaling pathways and mediated by a TIMP-3-sensitive metalloproteinase J Cell Biol 2000 148 4 811 824 10.1083/jcb.148.4.811 10684261 PMC2169376

[B42] Zellweger T Ninck C Mirlacher M Annefeld M Glass AG Gasser TC Mihatsch MJ Gelmann EP Bubendorf L Tissue microarray analysis reveals prognostic significance of syndecan-1 expression in prostate cancer Prostate 2003 55 1 20 29 10.1002/pros.10209 12640657

[B43] Juuti A Nordling S Lundin J Louhimo J Haglund C Syndecan-1 expression–a novel prognostic marker in pancreatic cancer Oncology 2005 68 2–3 97 106 15886501 10.1159/000085702

[B44] Barbareschi M Maisonneuve P Aldovini D Cangi MG Pecciarini L Angelo Mauri F Veronese S Caffo O Lucenti A Palma PD High syndecan-1 expression in breast carcinoma is related to an aggressive phenotype and to poorer prognosis Cancer 2003 98 3 474 483 10.1002/cncr.11515 12879463

[B45] Vassilakopoulos TP Kyrtsonis MC Papadogiannis A Nadali G Angelopoulou MK Tzenou T Dimopoulou MN Siakantaris MP Kontopidou FN Kalpadakis C Serum levels of soluble syndecan-1 in Hodgkin’s lymphoma Anticancer Res 2005 25 6C 4743 4746 16334170

[B46] Sebestyen A Berczi L Mihalik R Paku S Matolcsy A Kopper L Syndecan-1 (CD138) expression in human non-Hodgkin lymphomas Br J Haematol 1999 104 2 412 419 10.1046/j.1365-2141.1999.01211.x 10050727

[B47] Kyrtsonis MC Vassilakopoulos TP Siakantaris MP Kokoris SI Gribabis DA Dimopoulou MN Angelopoulou MK Pangalis GA Serum syndecan-1, basic fibroblast growth factor and osteoprotegerin in myeloma patients at diagnosis and during the course of the disease Eur J Haematol 2004 72 4 252 258 10.1046/j.0902-4441.2003.00205.x 15089762

[B48] Leivonen M Lundin J Nordling S von Boguslawski K Haglund C Prognostic value of syndecan-1 expression in breast cancer Oncology 2004 67 1 11 18 10.1159/000080280 15459490

[B49] Molica S Vitelli G Mirabelli R Digiesu G Giannarelli D Cuneo A Ribatti D Vacca A Serum levels of syndecan-1 in B-cell chronic lymphocytic leukemia: correlation with the extent of angiogenesis and disease-progression risk in early disease Leuk Lymphoma 2006 47 6 1034 1040 10.1080/10428190500470358 16840194

[B50] Aref S Goda T El-Sherbiny M Syndecan-1 in multiple myeloma: relationship to conventional prognostic factors Hematology 2003 8 4 221 228 10.1080/1024533031000153630 12911939

[B51] Joensuu H Anttonen A Eriksson M Makitaro R Alfthan H Kinnula V Leppa S Soluble syndecan-1 and serum basic fibroblast growth factor are new prognostic factors in lung cancer Cancer Res 2002 62 18 5210 5217 12234986

[B52] Thygesen K Alpert JS Jaffe AS Simoons ML Chaitman BR White HD Joint ESCAAHAWHFTFfUDoMI, Authors/Task Force Members C, Thygesen K, Alpert JS et al. Third universal definition of myocardial infarction J Am Coll Cardiol 2012 60 16 1581 1598 10.1016/j.jacc.2012.08.001 22958960

[B53] Thygesen K Alpert JS Jaffe AS Simoons ML Chaitman BR White HD Joint ESCAAHAWHFTFftUDoMI, Katus HA, Lindahl B, Morrow DA et al. Third universal definition of myocardial infarction Circulation 2012 126 16 2020 2035 10.1161/CIR.0b013e31826e1058 22923432

[B54] Thygesen K Alpert JS Jaffe AS Simoons ML Chaitman BR White HD Joint ESCAAHAWHFTFftUDoM: Third universal definition of myocardial infarction Eur Heart J 2012 33 20 2551 2567 10.1093/eurheartj/ehs184 22922414

[B55] Ray-Coquard I Cropet C Van Glabbeke M Sebban C Le Cesne A Judson I Tredan O Verweij J Biron P Labidi I Lymphopenia as a prognostic factor for overall survival in advanced carcinomas, sarcomas, and lymphomas Cancer Res 2009 69 13 5383 5391 10.1158/0008-5472.CAN-08-3845 19549917 PMC2775079

[B56] Porrata LF Ristow K Habermann TM Ozsan N Dogan A Macon W Colgan JP Witzig TE Inwards DJ Ansell SM Absolute monocyte/lymphocyte count prognostic score is independent of immunohistochemically determined cell of origin in predicting survival in diffuse large B-cell lymphoma Leuk Lymphoma 2012 53 11 2159 2165 10.3109/10428194.2012.690605 22551474

[B57] Koh YW Kang HJ Park C Yoon DH Kim S Suh C Kim JE Kim CW Huh J Prognostic significance of the ratio of absolute neutrophil count to absolute lymphocyte count in classic Hodgkin lymphoma Am J Clin Pathol 2012 138 6 846 854 10.1309/AJCPO46GFKGNXCBR 23161719

[B58] Koh YW Kang HJ Park C Yoon DH Kim S Suh C Go H Kim JE Kim CW Huh J The ratio of the absolute lymphocyte count to the absolute monocyte count is associated with prognosis in Hodgkin’s lymphoma: correlation with tumor-associated macrophages Oncologist 2012 17 6 871 880 10.1634/theoncologist.2012-0034 22588324 PMC3380887

[B59] De Giorgi U Mego M Scarpi E Giuliano M Giordano A Reuben JM Valero V Ueno NT Hortobagyi GN Cristofanilli M Relationship between lymphocytopenia and circulating tumor cells as prognostic factors for overall survival in metastatic breast cancer Clin Breast Cancer 2012 12 4 264 269 10.1016/j.clbc.2012.04.004 22591634

[B60] Watanabe K Yamashita Y Nakayama A Hasegawa Y Kojima H Nagasawa T Mori N Varied B-cell immunophenotypes of Hodgkin/reed-Sternberg cells in classic Hodgkin’s disease Histopathol 2000 36 4 353 361 10.1046/j.1365-2559.2000.00830.x 10759950

[B61] Carbone A Gloghini A Gaidano G Franceschi S Capello D Drexler HG Falini B Dalla-Favera R Expression status of BCL-6 and syndecan-1 identifies distinct histogenetic subtypes of Hodgkin’s disease Blood 1998 92 7 2220 2228 9746758

[B62] Buettner M Greiner A Avramidou A Jack HM Niedobitek G Evidence of abortive plasma cell differentiation in Hodgkin and reed-Sternberg cells of classical Hodgkin lymphoma Hematol Oncol 2005 23 3–4 127 132 16342298 10.1002/hon.764

[B63] Bai M Panoulas V Papoudou-Bai A Horianopoulos N Kitsoulis P Stefanaki K Rontogianni D Agnantis NJ Kanavaros P B-cell differentiation immunophenotypes in classical Hodgkin lymphomas Leuk Lymphoma 2006 47 3 495 501 10.1080/10428190500306784 16396774

[B64] Jones RJ Gocke CD Kasamon YL Miller CB Perkins B Barber JP Vala MS Gerber JM Gellert LL Siedner M Circulating clonotypic B cells in classic Hodgkin lymphoma Blood 2009 113 23 5920 5926 10.1182/blood-2008-11-189688 19188663 PMC2700327

[B65] Ibarrola De Andres C Toscano R Lahuerta JJ Martinez-Gonzalez MA Simultaneous occurrence of Hodgkin’s disease, nodal Langerhans’ cell histiocytosis and multiple myeloma IgA(kappa) Virchows Archiv Int J Pathol 1999 434 3 259 262 10.1007/s004280050338 10190308

[B66] Fujii H Iwai T Ueda Y Nakagawa H [Development of Hodgkin’s disease four years after autologous peripheral blood stem cell transplantation for multiple myeloma] [Rinsho ketsueki] Japan J Clin Hematol 2000 41 12 1267 1272 11201152

[B67] Newcom SR Gu L Transforming growth factor beta 1 messenger RNA in reed-Sternberg cells in nodular sclerosing Hodgkin’s disease J Clin Pathol 1995 48 2 160 163 10.1136/jcp.48.2.160 7745117 PMC502392

[B68] Kuittinen O Soini Y Turpeenniemi-Hujanen T Diverse role of MMP-2 and MMP-9 in the clinicopathological behavior of Hodgkin’s lymphoma Eur J Haematol 2002 69 4 205 212 10.1034/j.1600-0609.2002.02751.x 12431239

[B69] Zucker-Franklin D Grusky G Baez L Reed-Sternberg cells cultured from morphologically unidentifiable precursors in the blood of patients with Hodgkin’s disease Hematol Oncol 1983 1 2 127 138 6677562 10.1002/hon.2900010203

[B70] Sitar G Bianchi Santamaria A Rosti V Shaskin P Blago R Santamaria L Ascari E Giant cell formation in Hodgkin’s disease Res Immunol 1994 145 7 499 515 10.1016/S0923-2494(94)80069-3 7754197

